# Plant disease resistance is augmented in uzu barley lines modified in the brassinosteroid receptor BRI1

**DOI:** 10.1186/s12870-014-0227-1

**Published:** 2014-08-20

**Authors:** Shahin S Ali, Lokanadha R Gunupuru, G B Sunil Kumar, Mojibur Khan, Steve Scofield, Paul Nicholson, Fiona M Doohan

**Affiliations:** Molecular Plant-Microbe Interactions Laboratory, School of Biology and Environmental Science, University College Dublin, Dublin 4, Ireland; Department of Agronomy, USDA-ARS, Crop Production and Pest Control Research Unit and Purdue University, West Lafayette, IN 47907 USA; Department of Crop Genetics, John Innes Centre, Norwich Research Park, Norwich, NR4 7UH UK; SPCL, USDA/ARS Beltsville Agricultural Research Center, Beltsville, MD 20705 USA; Present address: Institute of Advanced Study in Science and Technology, Guwahati -35, India

**Keywords:** Disease resistance, Brassinosteroid, Uzu, BRI1, Fusarium

## Abstract

**Background:**

Brassinosteroid hormones regulate many aspects of plant growth and development. The membrane receptor BRI1 is a central player in the brassinosteroid signaling cascade. Semi-dwarf ‘uzu’ barley carries a mutation in a conserved domain of the kinase tail of BRI1 and this mutant allele is recognised for its positive contribution to both yield and lodging resistance.

**Results:**

Here we show that uzu barley exhibits enhanced resistance to a range of pathogens. It was due to a combination of preformed, inducible and constitutive defence responses, as determined by a combination of transcriptomic and biochemical studies. Gene expression studies were used to determine that the uzu derivatives are attenuated in downstream brassinosteroid signaling. The reduction of *BRI1* RNA levels via virus-induced gene silencing compromised uzu disease resistance.

**Conclusions:**

The pathogen resistance of uzu derivatives may be due to pleiotropic effects of BRI1 or the cascade effects of their repressed BR signaling.

**Electronic supplementary material:**

The online version of this article (doi:10.1186/s12870-014-0227-1) contains supplementary material, which is available to authorized users.

## Background

Brassinosteroids (BRs) are a family of hormones, involved in many cellular processes, including cell expansion and division, tissue differentiation, flowering, senescence and responses to abiotic stress [1,2]. BR hormones sequentially bind to the extracellular domains of the leucine rich repeat receptor BR Insensitive 1 (BRI1) and the co-receptor BRI1-associated Kinase 1 (BAK1) [3]. Transphosphorylation between BRI1 and BAK1 activates the former, which in turn leads to a downstream BR signaling cascade [4]. Nakashita et al. [5] were the first to demonstrate that BR hormones function in disease resistance in both tobacco and rice. Brassinolide (BL) is the end product of the BR biosynthetic pathway and in rice its application enhanced resistance to blast and bacterial blight diseases caused by *Magnaporthe grisea* and *Xanthomonas oryzae*, respectively. In tobacco, BL induced resistance to Tobacco Mosaic Virus, the bacteria *Pseudomonas syringae* pv. *tabaci* and the fungus *Oidium* sp. Resistance was not associated with accumulation of salicylic acid (SA) and systemic-acquired resistance (SAR) was not involved. Wang [6] reviewed BR-modulated plant responses to pathogens. In tobacco, virus-induced gene silencing of two homologs of BAK1 (NbSERK3A/B) enhanced susceptibility to the potato blight pathogen *Phytophthora infestans*, but not to its sister species *Phytophthora mirabilis* [7]. But BAK1/SERK3 is a multifunctional protein and at least some of its immune functions are independent of BR signaling [8]. It binds to the receptor for bacterial flagellin peptide, FLS2, eliciting PTI. As summarised by Wang [6], BR activation of BRI1 seems to have two opposite effects on BAK1-mediated FLS2 signaling, and the outcome seems to depend on the relative levels of BR, BRI1, and BAK1. BR signaling inhibits BIN2-mediated degradation of BRI1-EMS-suppressor 1 (BES1); BES1 is then produced and binds to a key defence regulator, AtMYB30, and together they function cooperatively to promote BR target gene expression [9]. BRs may also repress defence gene expression. The BR-activated transcription factor brassinazole-resistant 1 (BZR1) is able to repress the expression of genes such as *FLS2* and *SNC1* that are directly involved in defence against pathogens [8,10]. But, much remains to be determined regarding how the BR signaling cascade feeds into in plant disease resistance responses.

Uzu barley lines carry a mutation in a highly conserved residue (His-857 to Arg-857) in the kinase domain of the BR receptor protein BRI1 [11]. Recently Goddard et al. [12] and Chen et al. [13] showed that introgression of the uzu mutation into barley enhanced resistance to leaf blast disease caused by *Magnaporthe grisea,* take-all of roots caused by *Gaeumannomyces graminis* var. *tritici,* eyespot disease of stems caused by *Oculimacula* spp*.* and crown rot disease of the stem caused by *Fusarium* fungi. Here we investigate the resistance of uzu barley derivatives to more diseases and use a combination of transcriptomic and biochemical studies to determine how these uzu derivatives differ in defences and BR signaling as compared to their parental barley genotypes.

## Results and discussion

### Uzu enhances resistance to fungal and viral pathogens

**Figure 1:**
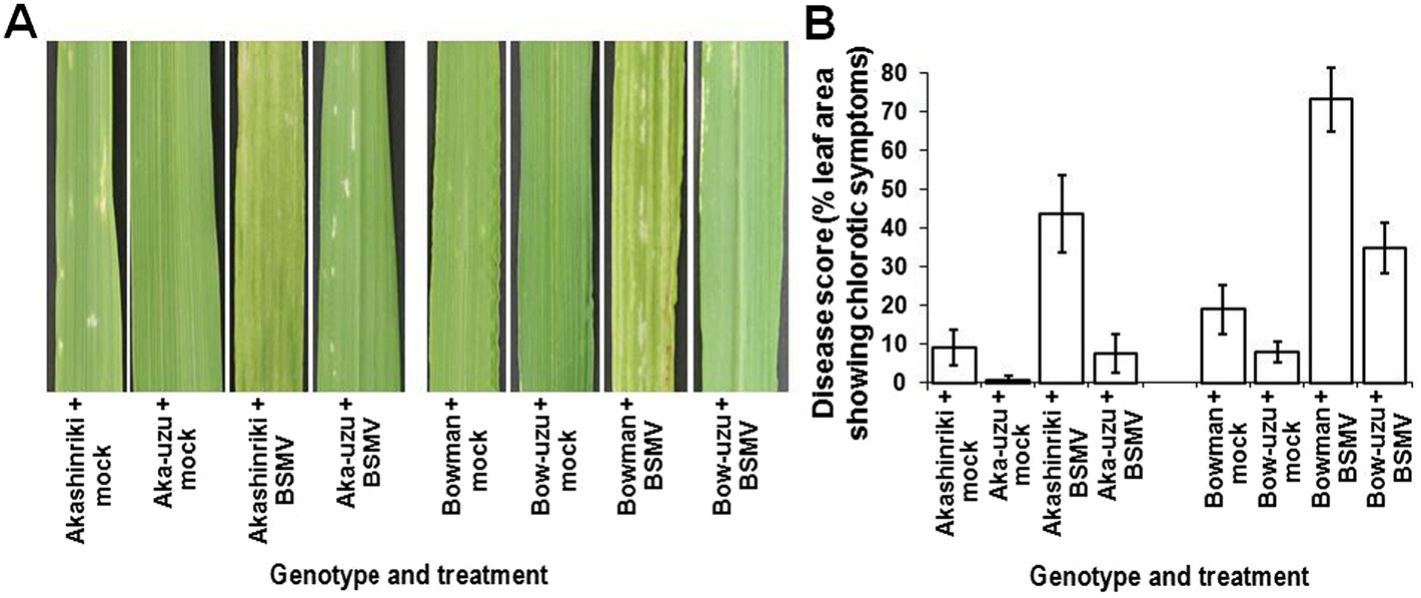
**Analysis of the phenotypic effect of Barley Stripe Mosaic Virus (BSMV) on seedling leaves of barley cultivars Akashinriki and Bowman and their uzu derivatives (Aka-uzu and Bow-uzu). (A)** Visualisation of the chlorotic stripes on the third leaves at 10 days post-infection. **(B)** Quantification of the percentage of leaf area showing chlorosis. Bars indicate standard error of mean (SEM) (LSD _0.05_ = 8.26).

**Figure 2:**
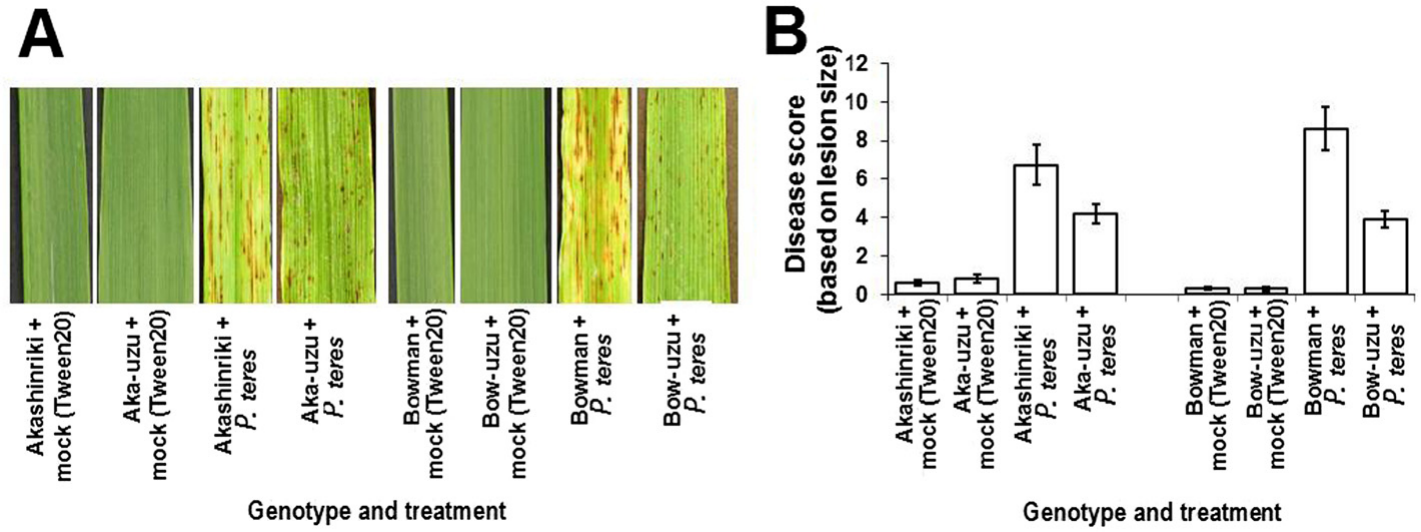
**Response of uzu derivatives of barley cultivars Akashinriki and Bowman (Aka-uzu and Bow-uzu) to net blotch disease caused by** ***Pyrenophora teres*** **f. sp.** ***teres*** **. (A)** Visualisation of the disease symptoms on seedling leaves at 10 days post-pathogen inoculation. **(B)** Disease scores based on lesion size [39] on both the second and third leaves at 10 days post-pathogen inoculation. Bars indicate standard error of mean (SEM) (LSD _0.05_ B = 2.54).

**Figure 3:**
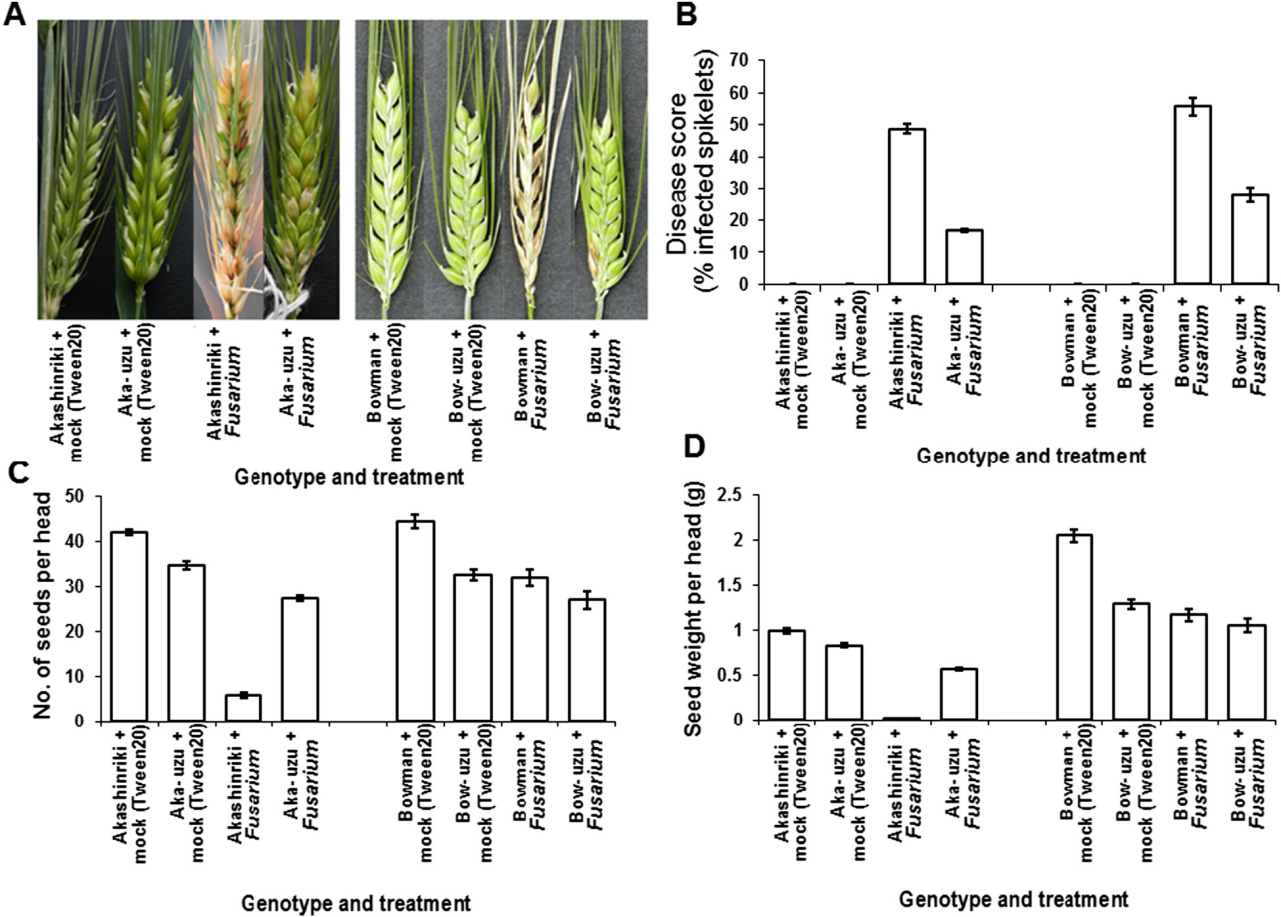
**Response of uzu derivatives of barley cultivars Akashinriki and Bowman (Aka-uzu and Bow-uzu) to Fusarium head blight (FHB) disease caused by** ***Fusarium culmorum*** **. (A)** Visualisation of the FHB disease symptoms at growth stage 80. **(B)** Disease symptoms at growth stage 80 were quantified as the percentage of infected spikelets per head. At harvest, the **(C)** number of grain per head and the **(D)** weight of the grain in each head were determined. Bars indicate standard error of mean (SEM) (LSD _0.05_ B = 1.3, C = 5, D = 0.11).

The semi-dwarf uzu derivatives of barley cvs. Akashinriki and Bowman were semi-dwarf derived via the introgression of a mutated *BRI1* gene from the old Japanese genotype Baitori 11 and subsequent backcrossing against the parent genotype [11]. Pathogenicity tests were used to compare the response of these two semi-dwarf uzu derivatives and their parental barley lines to the obligate pathogen Barley Stripe Mosaic Virus (BSMV), the necrotrophic net blotch pathogen *Pyrenophora teres* and the toxigenic hemibiotrophic fungus *Fusarium culmorum* that causes Fusarium head blight (FHB, also known as scab disease of cereals). Uzu derivatives were more resistant to all three pathogens as compared to the parental lines. When compared with their parents, uzu derivatives displayed significantly less symptoms of BSMV, net blotch and FHB disease (*P* ≤ 0.01; Figures 1, 2 and 3). The % of leaf area turned chlorotic due to BSMV was 80 and 52% less for the uzu derivatives of Akashinriki and Bowman, as compared to their respective parent lines (Figure 1). The severity of net blotch symptoms was 37% less in the Akashinriki-uzu derivative and 54% less in Bowman-uzu derivative, as compared to the respective parent lines (Figure 2). The FHB disease development (percentage of spikelets prematurely bleached) in the Akashinriki-uzu derivative was 65% less than that of the parent line. In the case of Bowman-uzu derivative, infected spikelets were 49% less as compared to the parent line (Figure 3B). FHB disease caused lower reductions in both grain number and weight in the uzu derivatives as compared to parent lines (Figure 3C and D). *Fusarium* fungi can also attack seedlings and leaves. Seedling blight studies revealed that, in response to *F. culmorum,* uzu derivative lines of both genotypes developed at least 41% less stem browning and 61% less leaf necrosis than parental lines (Additional file 1: Figures S1 and Figure S2) (*P* ≤ 0.01). Thus the uzu derivatives carry broad-spectrum disease resistance under controlled conditions, reaffirming and expanding the findings of Goddard et al. [12]. This study shows that the disease resistance of uzu derivatives is environment dependent, as the same uzu lines did not show resistance to initial FHB infection in recent UK field trials, albeit the disease pressure was relatively low [12]. Goddard et al. [12] recently presented evidence that the uzu derivatives of cvs. Akashinriki and Bowman had enhanced resistance to pathogens with a short biotrophic phase or a necrotrophic lifestyle, but not to the biotroph *Blumeria graminis* or to the leaf pathogen *Ramularia collo-cygni,* which has a long asymptomatic phase.

### Resistance components are constitutive, induced and derepressed

**Figure 4:**
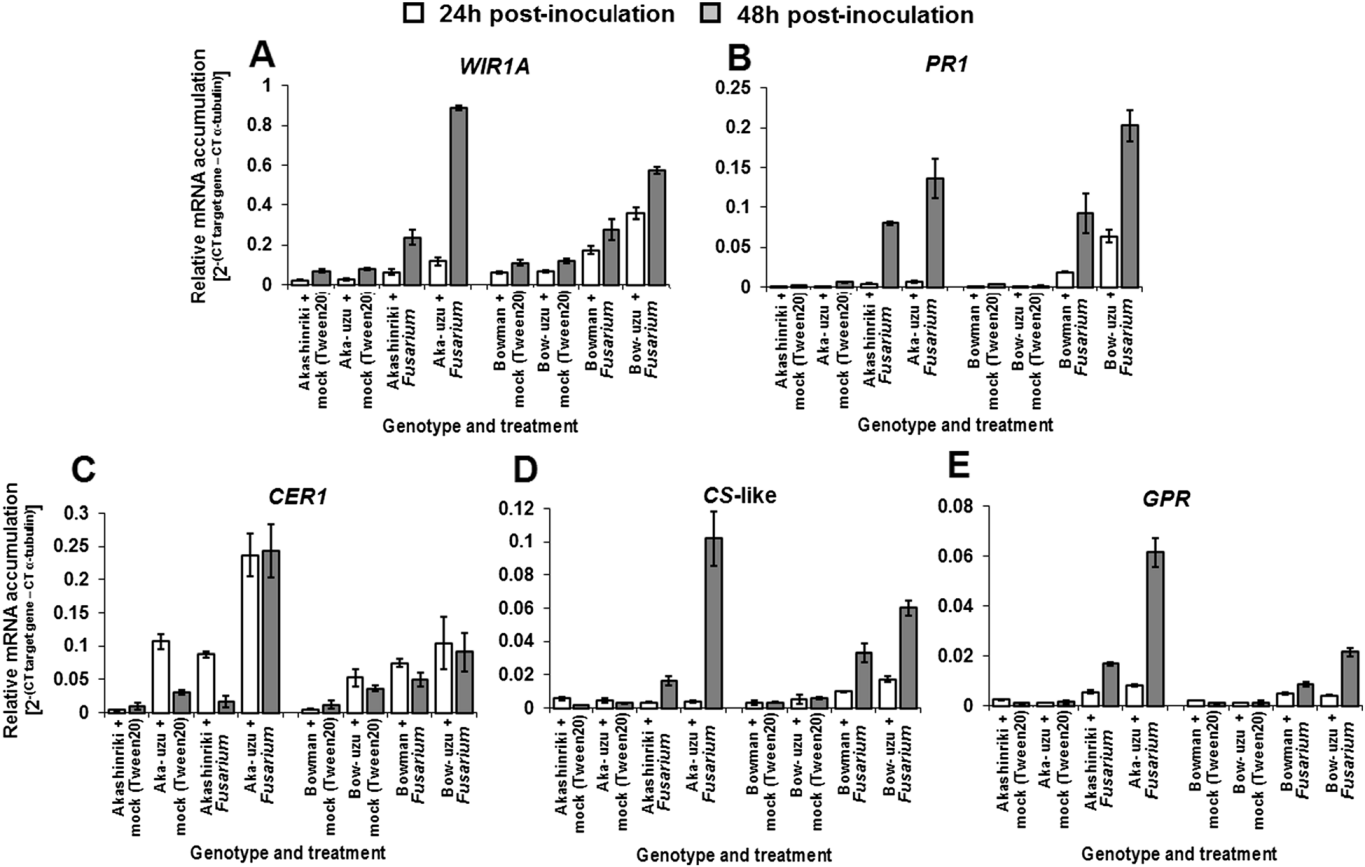
**Analysis of defence and cell wall biosynthesis gene transcript levels in the stem base of** ***Fusarium*** **-infected seedlings of barley cultivars Akashinriki, Bowman and their uzu derivatives (Aka-uzu and Bow-uzu), as determined by quantitative RT-PCR analysis.** Gene represented are **(A)** Pathogen-induced protein WIR1A (Contig2170), **(B)** Pathogenesis-related protein 1 (PR1) (Contig2210), **(C)** CER1 protein (Contig14570), **(D)** Cellulose synthase-like protein (CS-like) (Contig8067) and **(E)** Glycine-rich cell wall structural protein (GRP) (Contig3198). Bars indicate SEM (LSD_0.05_ A = 0.015 and B = 0.003, C = 0.015, D = 0.003, E = 0.001).

Microarray analysis of the early *Fusarium-*barley seedling interaction (24 h post-pathogen treatment of stems) indicated differential expression of putative defence genes in the Akashinriki-uzu derivative compared to the parental line. Comparison 1 showed that 155 transcripts were ≥ 1.5-fold differentially expressed in *F. culmorum-*treated Akashinriki-uzu derivative as compared to the *F. culmorum-*treated parent line (Additional file 2: Table S1 – excel file). A second microarray comparison delineated 126 transcripts as being pathogen-responsive in the uzu derivative (comparison of Akashinriki-uzu derivative +/- *F. culmorum*) (Additional file 3: Table S2 – excel file). Looking across both micorarray comparisons, a total of 44 transcripts were both more highly expressed in fungus-treated uzu as compared to fungus-treated parent and were also pathogen-responsive in the uzu derivative (Table 1). Many of these encode putative classical microbe-induced defence and PR proteins (Table 1). This includes putative chitinases, endo-1,3-beta-D-glucosidase and a β-1,3-glucanase, all of which act synergistically to degrade fungal cell walls [14]. Three putative thaumatin-like proteins were both fungal-induced and up-regulated in fungal treated uzu as compared to parent line (Table 1). Expression of a rice thaumatin-like protein was shown to enhance wheat resistance to FHB [15]. The transcription of indole glycerol phosphate and tryptophan synthase A is indicative of higher auxin production in uzu than the parent and in response to the pathogen. Tryptophan synthase B converts higher indole to tryptophan and this gene, although not pathogen-responsive, was up-regulated in uzu compared to the parent. Multilevel interactions between auxins and brassinosteroids have been reported; based on their study, Sakamoto et al. [16] suggested that auxins might control the degree of brassinosteroid perception by regulating the expression of the gene for brassinosteroid receptor. Auxin has been shown to enhance barley resistance to FHB disease [17], although the underpinning mechanisms were not elucidated. Quantitative RT-PCR validated that both a putative *WIR1A* and the classic *PR* gene *PR-1* are pathogen –responsive and more highly expressed in uzu derivatives than the parental lines Akashinriki and Bowman (Figure 4A & B). WIR1A has not been functionally characterised but it is thought to play a role in basal and non-host resistance [18]. WIR1A conferred wheat plants with resistance to the powdery mildew fungus *Blumeria graminis* [19] and two variants were mapped with the interval of a wheat genomic locus that confers FHB resistance [18].

**Table 1:** **Transcripts that were both pathogen-responsive and differentially regulated (≥1.5 fold) in the uzu derivative of barley cultivar Akashinriki-uzu, as compared to parent type barley line**

**Sequence Code** ^**#**^	**Microarray data comparisons**				Gene Ontology*	**BEST BLASTX** ^**±**^			
	**Fold induction (uzu vs Aka)** ^**¥**^	***P*** **-value**	**Fold induction (uzu + fungus vs uzu + no fungus)** ^**¥**^	***P*** **-value**		**Gene/protein ID**	**Annotation**	***E*** **value**	**Organism**
**Transcripts that were both pathogen up-regulated and up-regulated in uzu versus the parent**									
Contig5883_s	2.35282	9.89E-04	3.47814	4.86E-06	GO:0000162	AAM19104.1	Phosphoribosylanthranilate transferase	4.00E-35	*Oryza sativa*
Contig10483	1.77649	4.93E-04	2.03916	5.33E-05	GO:0016491	NP_177122.2	Acid phosphatase	7.00E-22	*Arabidopsis thaliana*
Contig12753	6.85586	3.48E-04	8.65154	1.60E-04	GO:0000166	BAB93292.1	Putative ABC transporter	4.00E-75	*Oryza sativa*
Contig10167	4.05623	1.60E-05	4.42652	1.38E-05	GO:0003700	BAB93351.1	Hypothetical protein B1131B07.13	6.00E-43	*Oryza sativa*
Contig11212	15.30721	8.05E-06	8.85902	2.01E-04	GO:0003824	AAL58118.1	Putative flavanone 3-hydroxylase	1.00E-72	Oryza sativa
HV_CEb0014M10r2	2.53882	4.62E-04	3.69271	1.20E-05	GO:0003824	AAF89745.3	Phosphatidic acid phosphatase beta	2.00E-15	*Vigna unguiculata*
Contig1179	3.15308	7.72E-04	3.69557	6.89E-04	GO:0005634	P05621	Histone H2B.2	5.00E-44	*Triticum aestivum*
Contig13350	8.37292	2.88E-08	38.39412	8.05E-12	GO:0005975	AAD28734.1	β-1,3-glucanase precursor	7.00E-65	*Triticum aestivum*
HV_CEb0022J21r2	4.86722	4.44E-04	10.55195	5.40E-05	GO:0006857	AAG46153.1	Putative peptide transporter	2.00E-32	*Oryza sativa*
HVSMEm0003C15r2_s	4.03305	1.11E-05	9.02364	1.73E-09	GO:0006952	A31800	Glucan endo-1,3-beta-D-glucosidase	2.00E-48	*Hordeum vulgare*
Contig10115	2.49729	9.01E-04	4.69131	1.10E-06	GO:0009058	NP_199633.1	Indole-3-glycerol phosphate synthase	1.00E-62	*Arabidopsis thaliana*
Contig18088	2.24148	9.93E-05	2.74884	9.52E-06	GO:0009536	S39045	Probable finger protein WZF1	2.00E-35	*Triticum aestivum*
Contig5542	2.61205	8.75E-04	2.91793	3.14E-04	GO:0009536	AAG42689.1	Putative tryptophan synthase α	1.00E-65	*Zea mays*
Contig21646	6.15205	6.18E-06	5.32979	4.06E-05	GO:0015297	BAC20746.1	Putative integral membrane protein	1.00E-26	*Oryza sativa*
Contig7898	2.74868	2.24E-04	2.68024	9.67E-04	GO:0016020	BAB64118.1	Hypothetical protein P0039A07.26	1.00E-62	*Oryza sativa*
Contig12237	3.62694	2.18E-08	7.23322	7.95E-12	GO:0016023	Q9M5X7	Nonspecific lipid-transfer protein precursor	2.00E-09	*Malus domestica*
Contig1636	2.83333	1.53E-05	3.82892	1.95E-07	GO:0016023	Q02126	Glucan endo-1,3-beta-glucosidase GIII	e-164	*Hordeum vulgare*
Contig1637	3.92582	1.23E-04	10.52758	2.30E-08	GO:0016023	D38664	Glucan endo-1,3-beta-D-glucosidase	e-162	*Hordeum vulgare*
Contig1637_s	3.72453	1.04E-04	6.92245	1.89E-07	GO:0016023	D38664	Glucan endo-1,3-beta-D-glucosidase	e-162	*Hordeum vulgare*
Contig1639	5.68725	2.62E-05	6.68401	8.82E-06	GO:0016023	S35156	β-glucanase	e-106	*Hordeum vulgare*
Contig2212_s	4.33587	3.48E-05	7.27081	3.89E-05	GO:0016023	P35793	Pathogenesis-related protein PRB1-3	8.00E-88	*Hordeum vulgare*
Contig26496	2.99884	5.20E-04	17.90724	4.22E-16	GO:0016023		No hit		
Contig2790_s	2.12349	1.71E-05	2.17092	5.04E-05	GO:0016023	AAK55325.1	Thaumatin-like protein TLP7	5.00E-74	*Hordeum vulgare*
Contig2990	2.32856	7.50E-04	4.22865	2.14E-06	GO:0016023	S48847	Chitinase	e-134	*Hordeum vulgare*
Contig3667_s	2.61309	8.97E-04	3.87741	9.78E-06	GO:0016023	T06179	MYB-related protein	e-164	*Hordeum vulgare*
Contig3947_s	2.40807	4.30E-04	3.94464	4.87E-07	GO:0016023	AAK55323.1	Thaumatin-like protein TLP4	5.00E-71	*Hordeum vulgare*
Contig4174	2.60929	2.83E-04	4.31725	6.89E-07	GO:0016023	S39979	Chitinase	1.00E-66	Oryza sativa
Contig6007	3.03741	2.95E-04	4.3984	1.99E-05	GO:0016023	BAB63883.1	Putative bromelain-like thiol protaease	7.00E-48	Oryza sativa
Contig2210/ Contig4054_s	2.816	3.17E-04	10.06151	8.75E-11	GO:0006952	Q05968	Pathogenesis-related protein 1	2E-80	*Hordeum vulgare*
Contig2550_x	2.11143	9.56E-04	3.1568	1.71E-05	GO:0016998	T06169	Pathogenesis-related protein 4	8.00E-77	*Hordeum vulgare*
Contig6576	2.21974	8.26E-04	4.15007	3.54E-05	GO:0016998	T06169	Pathogenesis-related protein 4	1.00E-50	*Hordeum vulgare*
Contig6576_s	2.92619	4.27E-04	5.06554	1.51E-06	GO:0016998	T06169	Pathogenesis-related protein 4	1.00E-50	*Hordeum vulgare*
Contig18116	3.68173	2.25E-04	6.95245	6.21E-07	GO:0043169	NP_181494.1	Glycosyl hydrolase family 17	1.00E-30	*Arabidopsis thaliana*
Contig2170/ Contig5974_s	18.26831	1.03E-11	11.48664	5.37-E06	GO:0016021	Q01482	Pathogen-induced protein WIR1A	3.00E-08	*Triticum aestivum*
HV_CEb0017C08r2	12.97883	4.83E-07	17.09899	3.83E-07	GO:0046274	AAK37826.1	Laccase	1.00E-19	*Pinus taeda*
Contig7354	2.06008	1.15E-04	2.04682	2.71E-04	GO:0050660	NP_567283.1	Protein id: At4g05020.1	4.00E-89	*Arabidopsis thaliana*
Contig13114	3.63475	9.89E-04	5.33272	8.45E-05	No hit	AAG21913.1	Putative cyanase	7.00E-08	Oryza sativa
Contig14625	3.09771	4.86E-04	3.05281	7.41E-04	No hit		No hit		
EBem10_SQ002_I10_s	3.3918	7.91E-08	3.93247	6.25E-08	No hit	AAK55326.1	Thaumatin-like protein TLP8	8.00E-04	*Hordeum vulgare*
EBro08_SQ012_C01	3.10739	4.52E-05	3.48265	2.89E-04	No hit		No hit		
HO04B16S	1.72257	6.40E-05	1.58429	0.00012	GO:0008152	BAB16426.1	Elicitor inducible gene product EIG-I24	2E-20	*Nicotiana tabacum*
Contig12169	1.9972	0.000228	1.62783	0.000548	GO:0016020	AAM93464.1	Unknown protein	9E-89	*Oryza sativa*
Contig6004	1.69354	1.41E-04	2.61521	5.12E-04	No hit	NP_564147.1	Unknown protein	2.00E-10	*Arabidopsis thaliana*
Contig2500	1.52109	0.000730	1.54984	0.000443	No hit	BAB07974.1	Unknown protein	1E-42	*Oryza sativa*
**Transcripts that were both pathogen down-regulated and down-regulated in uzu versus the parent**									
Contig7790	−4.25752	5.86E-05	−4.22732	8.49E-06	GO:0003676	NP_564325.1	Protein id: At1g29250.1	9.00E-44	*Arabidopsis thaliana*
Contig21684	−9.02614	7.66E-08	−2.09451	1.51E-04	GO:0016762	AAM62971.1	Putative xyloglucan endotransglycosylase	4.00E-34	*Arabidopsis thaliana*
Contig12965	−2.31955	9.43E-04	−7.42733	3.19E-06	GO:0005525	NP_192854.1	Translation initiation factor IF-2	1.00E-71	*Arabidopsis thaliana*
Contig4431_s	−2.82605	2.50E-10	−2.81901	9.86E-05	No hit	AAK44146.1	Unknown protein	1.00E-08	*Arabidopsis thaliana*
Contig17319	−1.84164	9.67E-04	−2.18675	1.31E-05	GO:0003723	BAC05662.1	Unknown protein	2.00E-40	*Oryza sativa*
Contig16536	−1.77974	6.09E-04	−1.91262	2.94E-04	GO:0003735	NP_189267.1	Unknown protein	1.00E-16	*Arabidopsis thaliana*

^#^Sequences of Affymetrix probes used in the microarray analysis were obtained using probe ID from http://www.plexdb.org.

^±^The homologs of barley transcript sequences from the barley database (http://www.plexdb.org) were determined by BLASTx analysis against the non-redundant protein database using the NCBI blast resource (www.ncbi.nlm.nih.gov).

^¥^Fold induction indicated up-regulation of gene in uzu compare to Akashinriki or uzu + fungus compare to uzu + no fungus.

*Gene ontology was analysed using the web-based tool agriGo http://bioinfo.cau.edu.cn/agriGO/.

**Figure 5:**
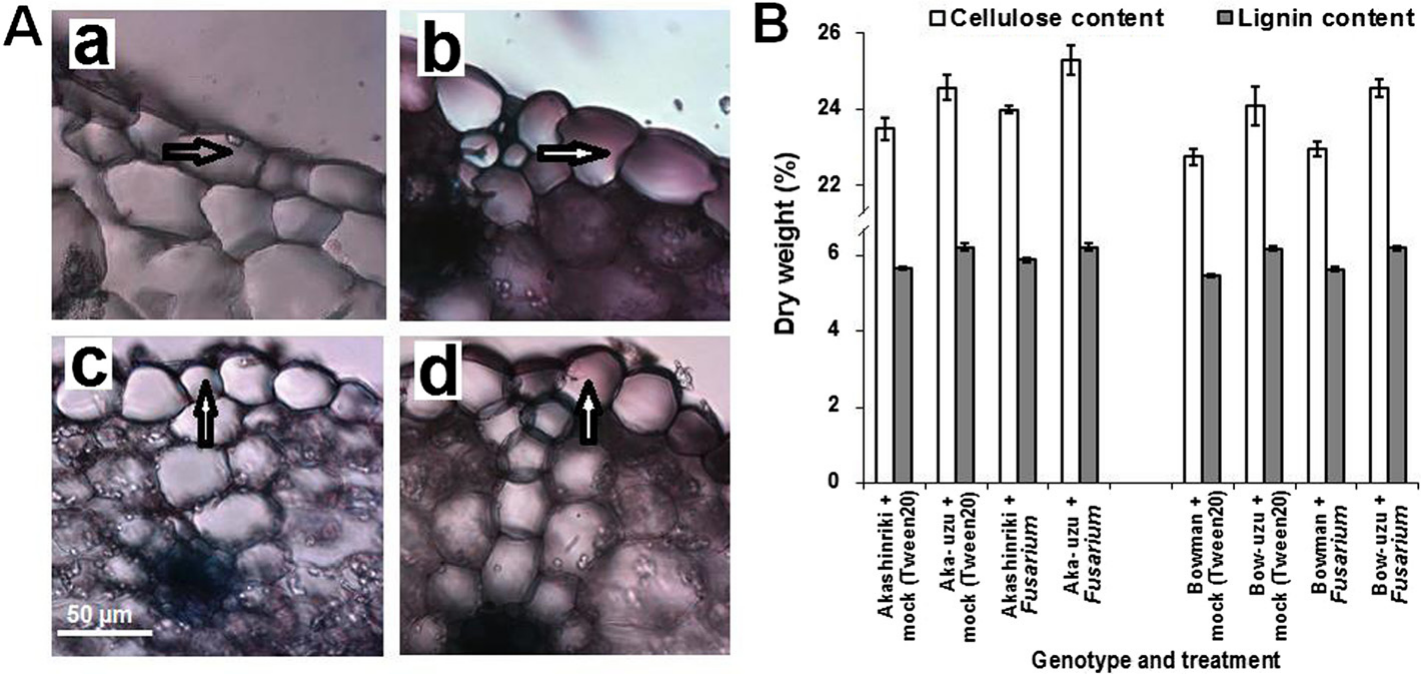
**The cell wall composition and epidermal cell morphology of leaves of barley cultivars Akashinriki and Bowman and their uzu derivatives (Aka-uzu and Bow-uzu). (A)** Twenty days post-stem base treatment the third leaves were removed and cleared for microscopic analysis (bar indicates 50 μm, arrows indicate epidermal cell layer) (**a**: Akashinriki, **b**: Akashinriki-uzu, **c**: Bowman and **d**: Bowman-uzu and **(B)** the remaining of the green plant tissue was pooled together and analysed for lignin and cellulose content. Bars indicate SEM (LSD _0.05_ cellulose = 1.12, lignin = 0.91).

Looking across both micorarray comparisons, a total of six trancripts were repressed in fungus-treated uzu as compared to the fungus-treated parent and were also pathogen-downregulated (Table 1). One of these encoded a putative xyloglucan endotransglycosylase (XET; Contig21684), which catalyses the remodeling of cell walls [20]. The plant cell wall presents a physical barrier to attack by pathogens and cellulose and lignin are amongst the wall constituents that contribute to defence [21,22]. A preliminary non-replicated microarray analysis at 48 h post-pathogen suggested that various genes involved in the production of wax (*CRE1*) and cell wall components (cellulose synthase and a glycine-rich cell wall structural protein) were pathogen-induced in the uzu derivative of cv. Akashinriki (data not shown). Transcriptomic, biochemical and microscopic analysis were used to investigate the cell size and wall composition in uzu derivatives and the parental lines. Quantitative RT-PCR analyses of uzu derivatives and parents showed that *CRE1*, cellulose synthase and a glycine-rich cell wall structural protein were highly expressed and more pathogen-responsive in uzu derivatives as compared to the parent lines Bowman and Akashinriki (Figure 4C-E). Biochemical analyses of non-infected uzu did not reveal pathogen-induced increases in the absolute amount of cellulose and lignin (*P* ≥ 0.05; Figure 5), but did confirm that the uzu derivatives have significantly higher cellulose and lignin contents and thicker cell walls than the parental lines (*P* ≤ 0.05).

**Figure 6:**
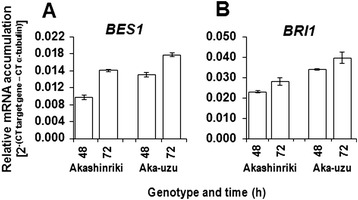
**Analysis of** ***BES1*** **and** ***BRI1*** **gene transcript levels in barley cultivar Akashinriki (Aka) and its uzu derivative (Aka-uzu), as determined by quantitative RT-PCR analysis.** Germinating seedlings were transferred to Hoaglands solution and the leaf samples are harvested after 48 and 72 hours for RNA extraction. **(A)** Brassinazole-resistant-2 (*BES1*) (Contig7854_at). **(B)** Brassinosteroid insensitive-1 (*BRI1*) (Gene id: AB109215.1). Bars indicate SEM (LSD_0.05_ A = 0.0003, B = 0.0048).

Further evidence for enhanced cell wall deposition in uzu derivatives was obtained via analysis of *BES1* transcription. BES1 can associate with upstream elements of most cellulose synthase genes and positively regulates cell wall synthesis [23]. Gene expression studies confirmed that seedling leaves from the Akashinriki-uzu derivative contained significantly more *BES1* transcript than those from the parent line Akashinriki (Figure 6A). The up-regulation of *BES1* transcription (Figure 6A), up-regulation of four chitinases of which two were pathogen-responsive (Table 1) and the enhanced ROS production during *F. culmorum* infection of Akashinriki-uzu derivative versus the parent line (Additional file 1: Figure S2B) are all indicative of the enhancement of SA-mediated defence responses [24,25]. *F. culmorum* is a hemibiotrophic pathogen, with a short biotrophic phase preceding necrotrophism [17]. Concordant with this it has been shown that the host SA dependent defence pathway is activated prior to jasmonic acid (JA) – dependent defence pathways during *Fusarium* infection [26].

**Figure 7:**
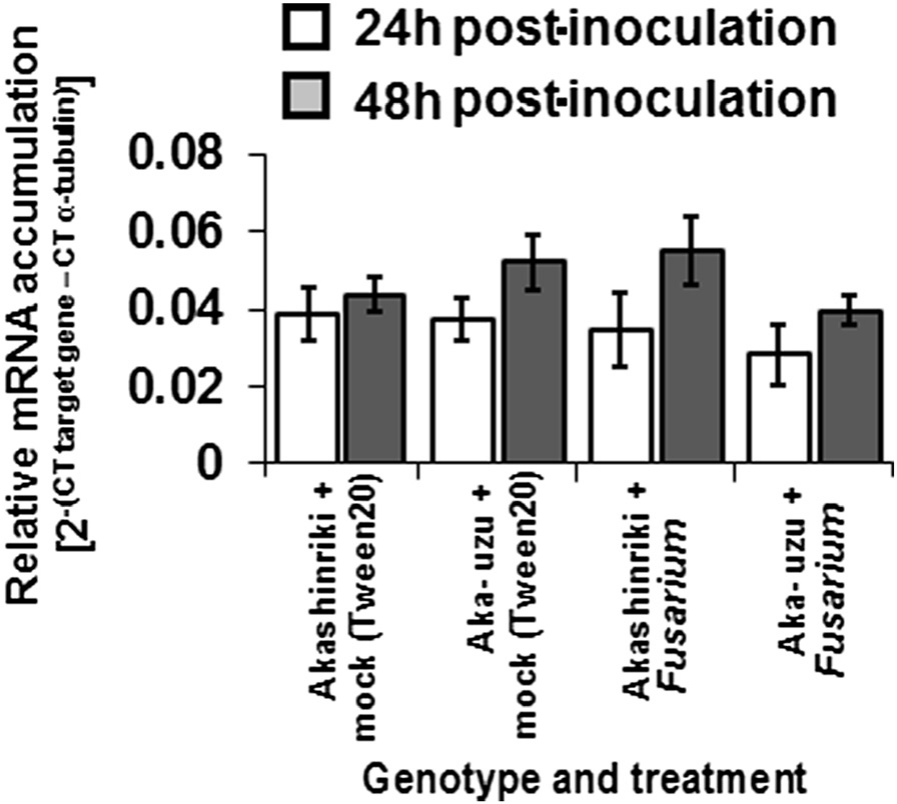
**Analysis of brassinazole-resistant 1 (** ***BZR1;*** **contig7854) gene transcript levels in the stem base of** ***Fusarium*** **-infected seedlings of barley cultivars Akashinriki, Bowman and their uzu derivatives (Aka-uzu and Bow-uzu), as determined by quantitative RT-PCR analysis.** RNA was extracted from stem bases tissue harvested at either 24 or 48 h post-fungal inoculation. Bars indicate SEM (LSD_0.05_ = 0.014).

Recent findings suggest that BR may antagonise plant defence signaling via activation of its transcriptional regulator BZR1. Upon activation by BR, BZR1 binds and represses the promoters of various defence-associated genes, including the flagellin receptor *FLS2* and the major *R* gene *SNC1* [10]. Interestingly we found that *BZR1* homolog of barley was slightly repressed in the Akashinriki-uzu-derivative at 48 h post *Fusarium* infection to levels similar to those found in healthy parental seedlings (Figure 7). This indicates that defences may be slightly derepressed in uzu derivatives.

### BR signaling is repressed in uzu

**Figure 8:**
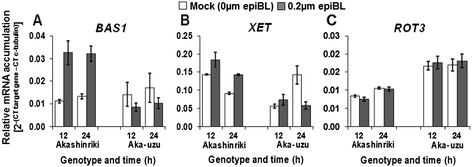
**Transcript levels of** ***BRI1*** **downstream and brassinosteroid metabolic genes in epibrassinolide (epiBL) treated seedlings of barley cultivars Akashinriki, Bowman and their uzu derivatives (Aka-uzu and Bow-uzu), as determined by quantitative RT-PCR analysis.** Germinating seedlings were treated with Hoaglands solution containing 5 μM of the brassinazole for 4 days and then with Hoaglands solution plus or minus 0.2 μm epiBL. Gene expression was quantified in samples harvested either 12 or 24 h post-treatment. Gene represented are **(A)** PHYB activation tagged suppressor 1 protein, *BAS1* (Contig3160), **(B)** *XET* (Contig5258) and **(C)** *ROT3* (Contig12042). Bars indicate SEM (LSD_0.05_ A = 0.0021, B = 0.016, C = 0.0021).

The literature suggests that the uzu derivative of barley is less responsive to BR [11]. We studied the effect of epibrassinolide (epiBL) treatment on the transcription of genes involved in BR signaling. At the mRNA level, the BR degradation enzyme gene *BAS1* and the downstream cell wall enzyme gene *XET1* are both positively regulated by BR [27,28], while the BR biosynthesis gene *ROT3* is down-regulated by hormone application [27]. Such was the effect of epiBL treatment on the transcription of *BAS1* and *XET1* in the parental cv. Akashinriki, while *ROT3* showed no effect of epiBL treatment (Figure 8). The epiBL application induced *BAS1* expression by three-fold by 12 h post treatment in cv. Akashinrki (*P* ≤ 0.05). But in the uzu derivative, the epiBL treatment had no major effect (*P* ≥ 0.05), the trend being for down-regulation of expression of *BAS1* in uzu in response to the hormone (Figure 8A). Similar results were obtained for *XET1* (Figure 8B), gene expression being significantly up-regulated in wild type seedlings but significantly down-regulated in uzu by epiBL treatment (*P* ≤ 0.05). We verified that (epiBL)-induced BR signaling was repressed in the Akashinriki-uzu derivative and thus the enhanced *BRI1* transcription in uzu does not lead to enhanced BR signaling. In both the wild type Akashinriki and the uzu derivative, there was no evidence of down- or up-regulation of BR biosynthesis, *ROT3* transcription being unresponsive to epiBL application (Figure 8C). This suggests that the enhanced resistance of uzu derivatives is not due to BR antagonising defence responses or driving enhanced negative cross talk with SA pathways [29].

### Virus-induced gene silencing of BRI1 comprises disease resistance in uzu

**Figure 9:**
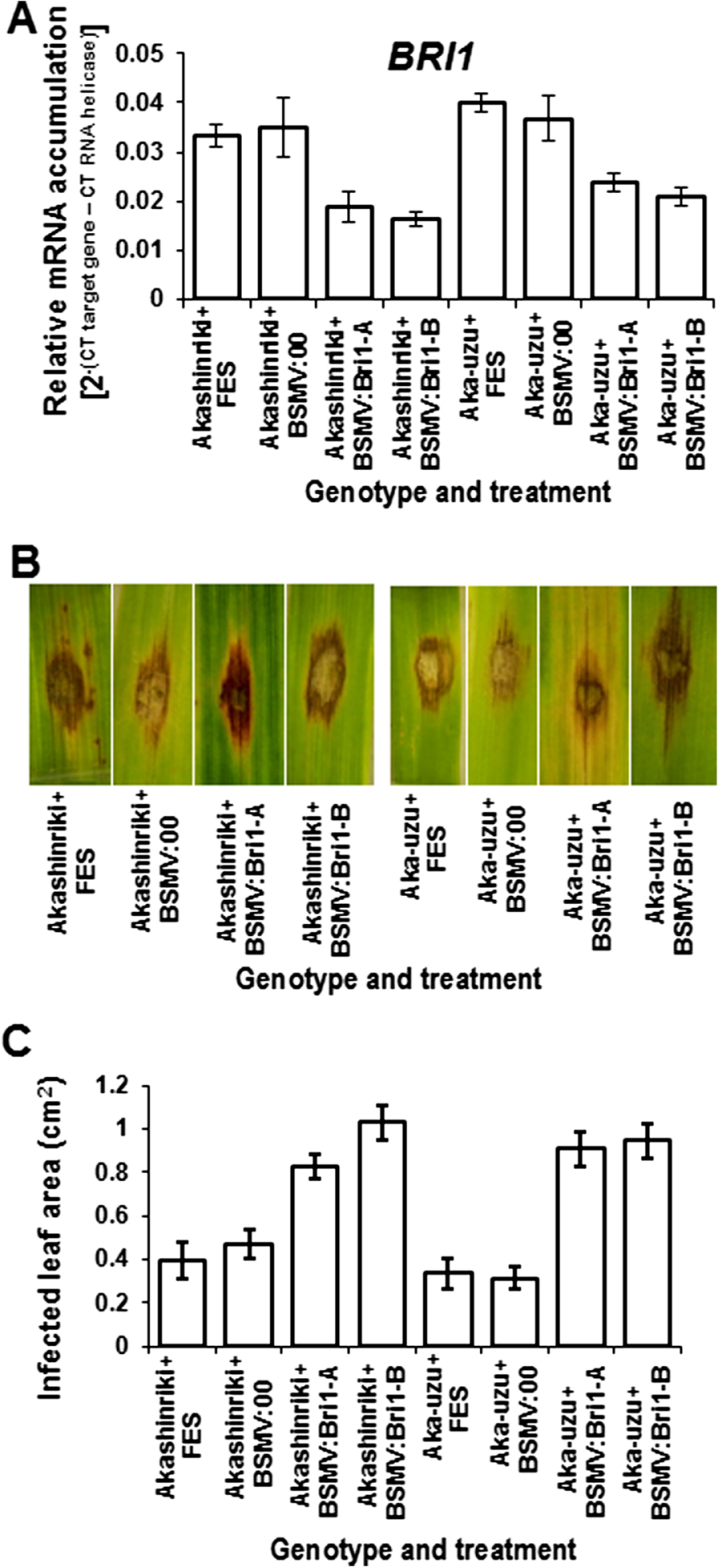
**Effect of virus-induced gene silencing (VIGS) of** ***BRI1*** **in the barley cultivar Akashinriki and its uzu derivatives on** ***Fusarium*** **infection of seedling leaves as assessed in a detached leaf experiment.** Plants were treated with viral application FES buffer, FES plus empty virus (BSMV:00) or FES plus virus targeting *BRI1* for silencing (BSMV:BRI1 & BSMV:BRI2 - two constructs containing independent fragments of the *BRI1* gene). At 14 days post VIGS treatment, 3^rd^ leaf was cleaved off for RNA extraction and used for gene expression studies and the detached leaf assay. **(A)** *BRI1* expression was quantified. **(B)** By 3 days after *F. culmorum* inoculation, infected area was more evident on gene-silenced as compared to empty virus treated samples of uzu and **(C)** this was quantified based on the pixel count using Image J software [41] (2000 pixel = 0.1 cm^2^). Bars indicate SEM (LSD _0.05_ A = 0.009, C = 0.254).

Pathogen-associated molecular pattern triggered immunity (PTI) is part of most broad-spectrum resistance/tolerance and BRs modulate PTI responses through both BAK1-dependent and -independent mechanisms [6,8]. Plant disease resistance depends on the relative levels of BR, and the receptor proteins BRI1 and BAK1. When the BRI1 level is low and the BAK1 level is not rate-limiting, increased BR signaling would enhance PTI signaling by providing active BAK1 [6]. However, in uzu derivatives, gene expression studies verified that *BRI1* transcript levels were high as compared to in the parental line Akashinriki (Figure 6B). Virus-induced gene silencing (VIGS) was performed in order to investigate the role of *BRI1* in disease resistance. To help rule out off-target effects in VIGS, two independent silencing treatments were performed, each targeting independent fragments of the *BRI1* gene (of 307 and 277 bp). Empty BSMV vector served as a negative control. VIGS of phytoene desaturase [30] served as a positive control in VIGS experiments, resulting in premature bleaching of both Akashinriki and uzu derivative plants (Additional file 1: Figure S3). The VIGS application buffer FES compromised leaf resistance to *F. culmorum.* The uzu/parental type differential observed without FES application (Additional file 1: Figure S2) was not observed in the leaf from plants treated with FES (Figure 9C). In both cases, the *Fusarium* disease was assessed on wounded detached sections of the third leaf, the only difference being that the FES applied to the first leaf in the VIGS experiments mostly likely induced a systemic wounding response in the VIGS experiments. It is possible, as suggested by Goddard et al. [12] that wounding, as occurs in VIGS treatment, may swamp the effect of the *BRI1*-related defence responses and may trigger JA signaling [31].

Quantitative RT-PCR gene expression studies validated that VIGS of *BRI1* was successful in both Akashinriki-uzu derivative and its parent (Figure 9A). The effect of fungal inoculation after VIGS-mediated silencing was assessed on the 3^rd^ leaf of VIGS-treated seedling. Reducing *BRI1* via VIGS enhanced *F. culmorum-*induced necrosis in leaves of both uzu derivative and parental type plants (Figure 9B). In the absence of *BRI1* gene silencing (BSMV:00 treatment), inoculated leaves showed a decrease in infected leaf area, relative to plants subjected to gene silencing (*P* < 0.01) (Figure 9C). These results suggest that a functional BRI1 is important for disease resistance. Goddard et al. [12] also reported that a T-DNA insertion in the 5′ untranslated region of *BRI1* homolog in *Brachypodium distachyon* resulted in a similar disease resistance response as observed in the uzu derivatives of barley. More insight into the importance of BRI1 in the disease resistance of uzu requires more in-depth studies of the receptor protein activity in barley derivatives and T-DNA mutants of *Brachypodium*.

## Conclusions

Uzu derivatives are mutated in the kinase domain of *BRI1* (this being the only mutation in a conserved domain of the protein). Xu et al. [28] showed that weak BRI1 mutants of Arabidopsis impaired in kinase activity still retain partial function in plant growth and development, indicating that BRI1 kinase activity is not essential for all activities of this receptor. Uzu resistance may be due to pleiotropic effects of BRI1 on another as yet uncharacterised pathway, indirect effects of the down-regulation of BR signaling or to genetic linkage between *BRI1* and a cosegregating resistance gene.

Though the majority of Japanese and Chinese semi-dwarf barley varieties carry the uzu mutation, the stress-intolerance of uzu barley [32] means that it may not be suited to all climatic conditions. A better understanding of the downstream defence mechanisms might highlight other targets that help control disease in a less environmentally dependent manner. To this end, we are investigating the disease resistance potential of a number of genes up-regulated in uzu in response to *F. culmorum.*

## Methods

### Plant and microbial material

The barley cultivars (cvs.) Akashinriki and Bowman and their uzu derivatives were kindly provided by Dr. K. Sato, Barley Germplasm Centre, Okayama University, Japan and the John Innes Centre. The uzu derivatives contain the *uzu* mutant version of the *BRI1* gene (GSHO1963), which was derived from Baitori 11, an old Japanese uzu barley. The *uzu* gene was introduced into barley cv Bowman and cv Akashinriki by sixtime and ninetime backcrossing, respectively [11]. The *F. culmorum* isolate used in this study was strain FCF 200. The fungus was stored at -70°C and, prior to use, was subcultured onto potato dextrose agar (PDA) (Difco, UK) plates and incubated at 25°C for 5 days. Fungal conidial inoculum was produced in mung bean broth as described previously [33]. *Pyrenophora teres* f. sp. *teres* strain N45 was stored at -70°C and, prior to use, were subcultured onto potato dextrose agar (PDA) (Difco, UK) plates and incubated at 25°C for 15 days. The plates containing fungal cultures were scraped and flooded with 0.2% Tween20 solution and the resulting conidial suspension was passed through a double-layer of cheesecloth to obtain the conidial inoculum. The tripartite genome of Barley stripe mosaic virus (BSMV) was maintained within plasmids and RNA generated by *in vitro* translation was used as inoculum, as previously described by Holzberg et al. [34].

### Fusarium head blight (FHB) experiments

All head blight experiments were conducted in glasshouse chambers at a temperature of 16 - 28°C. Barley cvs. Bowman and Akashinriki and their uzu derivatives were grown and, at mid anthesis, heads were treated with Tween20 (mock treatment) or *F. culmorum* conidia (1 × 10^6^ spores ml^−1^ 0.2% Tween20), as previously described [35]. Visual disease symptoms were recorded at GS 80 (start of dough development) [36] based on the percentage of bleached spikelets per head. Treated heads were harvested at growth stage 99. The number of seeds per head and seed weight (g) per head were recorded. Each treatment combination was applied to sixteen plants (2 heads per plant) and the experiment was conducted thrice (February to April 2010, Feb to April 2012 and March to May 2012) in different glass house chambers in a randomized layout.

### Fusarium seedling blight (FSB) experiments

Seeds of barley cvs. Bowman and Akashinriki and their uzu derivatives were germinated, grown in a 6 cm diameter pot containing John Innes compost No. 2 (Westland Horticulture, Dun- gannon, UK). The plants were grown in a climate-controlled growth room with day/night temperatures of 20/12°C, a 12 h light period (700 μmol m^−2^ s^−1^) and constant humidity of 85%. Stem bases of 10-day-old seedlings were treated as previously described [37] with 400 μl of either a *F. culmorum* conidial suspension (1 × 10^6^ spores ml^−1^ 0.2% Tween20) in 1% agar (Difco Laboratories, Detroit, MI) or 0.2% Tween20 in 1% agar (mock treatment). The stem base samples (4 cm) were harvested at 15 days post-fungal treatment. Seedling blight stem base disease symptoms were scored as the product of lesion length (cm) and lesion colour (lesion colour scale: 0, no disease; 1, very slight brown necrosis; 2, slight/moderate brown necrosis; 3, extensive brown necrosis; 4, extensive black necrosis) [38]. This experiment was conducted thrice, and each time it included three replicate pots (each containing two plants) per treatment combination, arranged in a randomised layout. To analyze gene expression in response to *F. culmorum* seedling inoculations, similar experiments were conducted on cv. Akashinriki and its uzu derivative, except that samples were flash-frozen in liquid nitrogen and stored at -70°C prior to RNA extraction. Three independent experiments with six replicate pots (each containing two plants) per treatment combination were conducted for microarray analysis (24 h harvest time point) and two independent experiments with three replicate pots (each containing two plants) per treatment combination were conducted for quantitative RT-PCR analysis (24 and 48 h harvest time points).

### Net blotch experiments

Seeds of barley cvs. Bowman and Akashinriki and their uzu derivatives were germinated and grown as described above for FSB studies. Foliage of 15 day old barley seedlings were sprayed to runoff with a conidial suspension (4 × 10^4^ spores ml^−1^ 0.2% Tween20 of *P. teres* f. sp. *teres* strain N45 or 0.2% Tween20 (mock treatment). The disease score was calculated based on the average infection phenotype of the second and third leaves scored using a 1-10 scale [39]. Results were based on three experiments, all of which included 10 replicate pots (each containing 2 plants) per treatment combination.

### Barley stripe mosaic virus experiment

Seeds of barley cvs. Bowman and Akashinriki and their uzu derivatives were germinated and grown as described above for FSB studies, except that experiments were conducted in a contained glasshouse where the temperature was a constant 24°C, and supplemental lighting of 700 μmol m^−2^ s^−1^ for 16 h per day was provided. The first leaf of 10 day old seedlings was rub-inoculated with BSMV RNA or with FES buffer (mock treatment) following the protocol described by Scofield et al. [30]. Disease on the third leaf was assessed at 14 days post inoculation, based on the percentage leaf area showing chlorosis. Results were based on two experiments, all of which included 10 replicate pots (each containing 2 plants) per treatment combination.

### Detached leaf assay

Seedlings of barley cvs. Bowman and Akashinriki and their uzu derivatives were germinated and grown as described above for FSB studies. The third leaves were harvested from 20 day-old seedlings. Leaf sections (5 cm) were placed on filter paper soaked in 0.08% benzimidazole solution and the upper epidermal layer in the centre of the leaf was surface-wounded by making 4-5 holes using a sterile needle. The damaged leaf area was treated with 5 μl of either 0.2% Tween20 (mock treatment) or *F. culmorum* conidia (1 × 10^6^ spores ml^−1^ 0.2% Tween20). Leaf samples were photographed 72 h post-inoculation and subjected to 3,3′-diaminobenzidine (DAB) staining to detect ROS formation [40]. Leaf samples were placed in a solution of 1 mg ml^−1^DAB, and collected for photography after 8 h. Infected leaf area was measured based on the pixel count using Image J software [41] and the ROS formation was measured based on the total pixel count from 0-100 at a scale of 0-250 and converted to leaf area (2000 pixel = 0.1 cm^2^).

### Seedling composition and epidermal cell morphology

Seedlings of barley cvs. Bowman and Akashinriki and their uzu derivatives were germinated and grown as described above for FSB studies. Stem bases of 10 day old seedlings were treated with either Tween20 in 1% agar or *F. culmorum* conidia (1 × 10^6^ spores ml^−1^ 0.2% Tween20) and 1% agar. After twenty days, seedlings were cut above the stem base. Leaf sections were treated with absolute alcohol over night at 60°C to remove the chlorophyll and transvers sections were prepared for microscopic study. The remaining green plant material was oven dried for 7-10 days at 55°C and subjected to cellulose and lignin estimation using the methods of Ali et al. [42]. Cellulose and lignin content were determined for three sub-samples per cultivar and were expressed as a percentage of dry weight. Results were based on three experiments, all of which included 10 replicate pots (each containing 2 plants) per treatment combination.

### Epibrassinolide (epiBL) treatment experiment

Barley seeds were surface-sterilized with 2% bleach, and kept on Whatman paper in dark at 4°C for 2 days for synchronisation of seed growth. After two days the petri plates were transferred to 25°C degrees in darkness. Three-day-old germinating seedlings were transferred to hydroponic system containing Hoagland’s solution supplemented with 5 μM of Brassinazole (BRZ) in order to inhibit endogenous BR production. The plants were placed in an incubator with continuous light of 700 μmol m^−2^ s^−1^ at 25°C. After four days the BRZ solution was replaced by Hoagland’s medium containing 0.2 μM epiBL (in 70% ethanol) and 70% ethanol (mock treatment). Samples were collected from the mock and epiBL treated plants at 12 and 24 h post-treatment and flash frozen in liquid nitrogen prior to RNA extraction.

### RNA extraction

Total RNA was extracted from the stem base samples using the protocol described by Chang et al. [43]. RNA extracts were DNase1-treated according to manufacturer’s instructions (Invitrogen corp., Carlsbad, CA) and resuspended in diethyl pyrocarbonate (DEPC)-treated water. The quantity of RNA in samples was assessed using an Eppendorf Biophotometer (Eppendorf AG, Hamburg, Germany), according to manufacturer instructions. RNA quality of samples was assessed by estimating the RNA integrity number (RIN) [44,45] which averaged > 8, indicating high quality RNA.

### Microarray analysis

Microarray analysis was used to analyse the early effects of *F. culmorum* on the transcriptome of seedlings of Akashinriki and its uzu derivative (24 h post-fungal inoculation). Microarray production, hybridization, and data analysis were performed following the minimum information about a microarray experiment (MIAME) guidelines for international standardization and quality control of microarray experiments [46]. Microarray analysis was conducted using three composite samples per treatment; composite samples were produced by pooling equal amounts of the total RNA (1 μg) from the five replicate samples per treatment per experiment per time point. Total RNA (1 μg) from each sample was converted to double-stranded cDNA with the Bioarray™ single-round RNA amplification and labeling kit (Enzo life sciences, PA, USA). After second-strand synthesis, the cDNA was purified with the cDNA purification kit (Enzo life sciences). The resulting double-stranded DNA was then used to generate multiple copies of biotinylated cRNA by *in vitro* transcription with the bioarray™ highyield™ RNA transcript labeling kit (Enzo life sciences). The A_260/280_ ratio and yield of each of the cRNAs were determined and the quality of these samples was assessed using an Agilent bioanalyzer (Agilent Technologies, Palo Alto, CA, USA**)** and all exceeded the RNA integrity number threshold of 8. Biotinylated cRNA (10 μg) spiked with *biob, bioc, biod* and *cre* (hybridization controls) was hybridized to the Affymetrix barley GeneChip array (Affymetrix, Inc. CA, USA) for 16 hours at 45°C. Following hybridization, all arrays were washed and stained in an Affymetrix GeneChip fluidics station. Stained arrays were scanned with an Affymetrix GeneChip® scanner 3000 (Affymetrix, Inc. CA, USA). Quality checks and data analyses were carried out using affymetrix GeneChip operating software (gcos) and quality reporter. The array data was normalised per chip and per gene. Per chip normalisation is carried out to the median. For per gene normalisation, comparisons were conducted for the three expression values obtained across three biological replicates. Two comparisons were conducted; firstly we compared gene expression in the uzu derivative + fungus, versus Akashinriki + fungus; secondly we compared expression in uzu – fungus versus uzu + fungus. A list of significant probes were generated using a student t-test with a fold-change threshold of ≥ 1.5 and ≤ -1.5 higher transcript with a *P-value <* 0.0001*.*

### Sequence analysis

For each probe set, annotations of associated genes/gene homologs were obtained directly from the Affymetrix website (https://www.affymetrix.com/analysis/netaffx/showresults.affx) or by BLASTx analysis against the non-redundant protein database [47] using the National Center for Biotechnology Information (NCBI) blast resource (www.ncbi.nlm.nih.gov). The cut-off value of 10^−15^ was used as a threshold for the expectation scores (e values), and only homologies with an e-value of less than the threshold were regarded as significant. Whenever, the two descriptions disagreed, the BLASTx description was selected.

### Quantitative RT-PCR analysis

Quantitative RT-PCR was used to analyse the expression of transcripts of interest. Reverse transcription (RT) of 1 μg total RNA was conducted as described by Ansari et al. [48], except that the primer used was oligo dT_12-18_ (Invitrogen). RT products (25 μl) were diluted to 200 μl and 2.5 μl was PCR-amplified in a 25 μl volume reaction containing 12.5 μl Premix *Ex Taq*™ (Perfect Real Time) (Takara, Japan) and 100 nM each of forward and reverse transcript-specific primers (Additional file 4: Table S3). PCR reactions were conducted in a Stratagene Mx3000™ quantitative RT**-**PCR machine (Stratagene, USA) and the programme consisted of 1 cycle of 95°C for 10s, 40 cycles of 95°C for 5 s, 60°C for 30s and 1 cycle of 95°C for 60s. Data were analysed using Stratagene Mx3000™ software (Stratagene, USA). The housekeeping gene used for normalisation of quantitative RT-PCR data was α-tubulin (Affymetrix Contig127_s_at); real-time quantification of target gene and of the housekeeping gene was performed in separate reactions. The threshold cycle (CT) values obtained by quantitative RT-PCR were used to calculate the accumulation of target gene (relative mRNA accumulation), relative to α-tubulin transcript, by 2^^-∆∆Ct^ method, where ∆∆Ct = (Ct target gene - Ct α-tubulin) [49]. Results were based on the average obtained for at least two replicate quantitative RT-PCR reactions per sample.

### Virus-induced gene silencing (VIGS)

The barley stripe mosaic virus (BSMV)-derived VIGS vectors used in this study consisted of the wild type BSMV ND18 α, β and γ tripartite genome [30,34]. The VIGS fragments and the quantitative RT-PCR assay used to validate VIGS targeted *HvBri1* (AB109215.1) on the 3HL chromosome of barley genome, as determined by BLAST analysis against the IPK barley genome database (results not shown). Two independent gene fragments were used for VIGS of *HvBri1* and these were amplified from genomic DNA of barley cv. Akashinriki using the primers *HvBri1*A-F/R or *HvBri1*B-F/R (Additional file 4: Table S4). PCR reactions were performed with 30 ng of barley genomic DNA, 1 mM each of forward and reverse fragment-specific primers (Additional file 4: Table S4) in a 10 μl reaction containing 0.5U Taq DNA polymerase and 1× PCR buffer (Invitrogen, UK), 1.5 mM MgCl_2_, and 125 mM of each dNTP. PCR reactions were conducted in a Peltier thermal cycler DNA engine (MJ Research, USA) and the PCR program consisted of an initial denaturation step at 94°C for 2 min, 30 cycles of denaturation 94°C for 30 s, annealing at 60°C for 30 s, extension at 72°C for 45 s and a final extension step at 72°C for 5 min. The amplified silencing fragments were cloned into the pGEM-T vector (pGEM-T Easy cloning kit; Promega, UK). The pGEM-T vectors carrying the silencing fragments were then digested with Pac1 and Sma1. The inserts were purified by gel extraction and then cloned into Pac1 and Sma1 digested γ RNA vector, pSL038-1 [30]. The pSL038-1 plasmids harbouring the silencing fragments were sequenced by Macrogen Inc. (Korea) using the vector-specific primers pGamma-F/R (Additional file 4: Table S4). A BSMV γ RNA construct containing 185 bp-fragment of the barley phytoene desaturase (*PDS*) gene was used as a positive control for VIGS and has been previously described [30]. The plasmids that contain the BSMV genome α and γ constructs with silencing fragments for *PDS* and *HvBri1*A or *HvBri1*B were linearised with *Mlu*I. The plasmid with BSMV β genome was linearised with *Spe*I. Capped *in vitro* transcripts were prepared from the linearised plasmids using the mMessage mMachine T7 *in vitro* transcription kit (Ambion, Austin, TX) following the manufacturer’s protocol. The first leaves of 10-day-old seedlings were rub-inoculated with BSMV constructs following the protocol described by Scofield et al. [30]. Rub inoculations were done with 1:1:1 mixtures of the *in vitro* transcripts of BSMV α, β and γ RNA (BSMV:00) or derivatives of the γ RNA that contained barley *PDS* (BSMV:PDS), *HvBri1*A or *HvBri1*B fragments. After 14 days the 3^rd^ leaf is taken and made into three segments, one segment flash frozen in liquid N_2_ and stored at -70°C prior to RNA extraction. Gene silencing was quantified using primers specific to *BRI1* and relative to that of the RNA helicase housekeeping gene [50]. The remaining 2 sections were used for the detached leaf *Fusarium* assay as described above (using a total of 8 leaf sections per *Fusarium* and 8 per mock Tween20 treatment for each silencing construct). After 3 days symptoms were observed and recorded. The VIGS experiment was conducted three times.

### Statistical analysis

Normal distribution of data sets was determined using the Ryan Joiner test [51] within Minitab (Minitab release 13.32^©^, 2000 Minitab Inc.). Non-normally distributed data sets were transformed to fit a normal distribution using the Johnson transformation [51] within Minitab (Minitab release 13.32^©^, 2000 Minitab Inc.). The homogeneity of data sets across replicate experiments was confirmed by two-tailed correlation analysis (non-normal data: Spearman Rank; normal data: Pearson product moment) conducted within the Statistical Package for the Social Sciences (SPSS 11.0, SPSS Inc.) (*r* ≥ 0.798; *P =* 0.01) [52]. Therefore, data sets from the replicate experiments were pooled for the purposes of further statistical analysis. The significance of treatment effects was analysed within Statistical Package for the Social Sciences (SPSS 11.0, SPSS Inc.) by either (i) normally distributed data - one-way ANOVA with Post Hoc pair wise Least Significance Difference (LSD) comparisons (P = 0.05), or (ii) non-normally-distributed data - the Kruskal-Wallis H test [52].

### Availability of supporting data

The data sets supporting the results of this article are included within the article and its supplementary files.

## Additional files

Additional file 1: Figure S1. Response of uzu derivatives to Fusarium seedling blight disease. **Figure S2.** Leaf response of uzu derivatives to *Fusarium culmorum.* **Figure S3.** Effect of virus-induced gene silencing (VIGS) of phytoene desaturase (PDS) on the phenotype of leaves of barley cultivar Akashinriki and its uzu derivative. Additional file 2: Table S1. Differentially regulated (≥1.5 fold) transcripts between brassinosteroid-insensitive barley line uzu and wild type parent Akashinriki in response to Fusarium seedling blight. Additional file 3: Table S2. Transcripts differentially regulated (≥1.5 fold) in the uzu derivative of cv. Akasinriki in response to Fusarium seedling blight. Additional file 4: Table S3. Primers used for real time quantitative RT-PCR analysis. **Table S4.** Primers used for VIGS.
