# Supplementary material for: Plant disease resistance is augmented in uzu barley lines modified in the brassinosteroid receptor BRI1
Source: BMC Plant Biol. 2014 Aug 20;14:227. doi: 10.1186/s12870-014-0227-1 (PMC4158134; doi:10.1186/s12870-014-0227-1)
Supplement: Additional file 1: Figure S1. — Response of uzu derivatives to Fusarium seedling blight disease. Figure S2. Leaf response of uzu derivatives to Fusarium culmorum. Figure S3. Effect of virus-induced gene silencing (VIGS) of phytoene desaturase (PDS) on the phenotype of leaves of barley cultivar Akashinriki and its uzu derivative. [file 12870_2014_227_MOESM1_ESM.docx]

**SUPPLEMENTARY FIGURES**

**Plant disease resistance is augmented in uzu barley lines modified in the brassinosteroid receptor BRI1**

Shahin S. Ali^1,4©†^, Lokanadha R. Gunupuru^1†^, G.B. Sunil Kumar^1^, Mojibur Khan^1±^, Steve Scofield^2^, Paul Nicholson^3^ and Fiona M. Doohan^1©^

^1^Molecular Plant-Microbe Interactions Laboratory, School of Biology and Environmental Science, University College Dublin, Dublin 4, Ireland.

^2^USDA-ARS, Crop Production and Pest Control Research Unit and Purdue University, Department of Agronomy, 915 West Street, West Lafayette, IN 47907-2054, USA

^3^Dept. of Crop Genetics, John Innes Centre, Norwich Research Park, Norwich NR4 7UH, UK.

^4^SPCL, USDA/ARS Beltsville Agricultural Research Center, MD, USA.

^±^Present address: Institute of Advanced Study in Science and Technology, Guwahati -35, India.

^†^Equal contributors

Corresponding author:

Email: shahinsharif.ali@gmail.com, fiona.doohan@ucd.ie

Fax: 0035317161102

Phone: 0035317162248


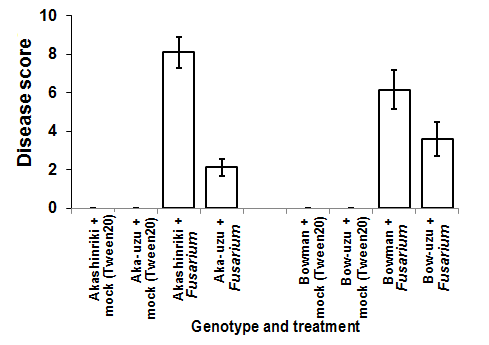


**Figure S1** Response of uzu derivatives to Fusarium seedling blight disease. Stem bases of 10-day-old seedlings of barley cultivars Akashinriki and Bowman and their uzu derivatives (Aka-uzu and Bow-uzu) were treated with either *Fusarium culmorum* conidia or mock Tween20 treatment. Fifteen days post-stem base treatment, the visual disease symptoms were assessed. Disease score was the product of lesion length (cm) by lesion colour (lesion colour scale: 0, no disease; 1, very slight brown necrosis; 2, slight/moderate brown necrosis; 3, extensive brown necrosis; 4, extensive black necrosis) [38]. Bars indicate SEM (LSD _0.05_ = 1.46).

**
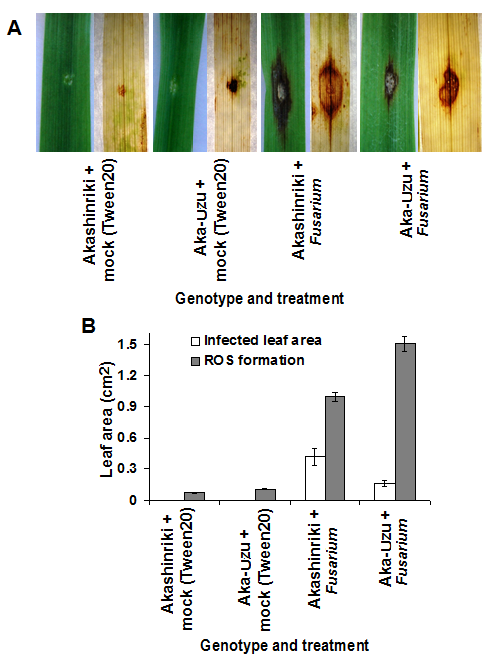
**

**Figure S2** Leaf response of uzu derivatives to *Fusarium* *culmorum.* Leaf sections from the third leaves of barley cultivars Akashinriki and Bowman and their uzu derivatives (Aka-uzu and Bow-uzu) were wounded and treated with either conidia of *F. culmorum* or mock Tween20 treatment and leaves were examined visually or assessed for reactive oxygen species (ROS) production at 72h post-treatment. **(A)** Visualization of symptoms (green leaves) and ROS (cleared leaves). **(B)** Quantification of infection and ROS formation based on lesion size. Necrotic leaf area and ROS-positive area was calculated based on the pixel count using Image J software [41] and the ROS formation was measured based on the total pixel count from 0-100 scale and converted to leaf area (2000 pixel = 0.1 cm^2^). Bars indicate SEM (LSD _0.05_ B = 0.0362).


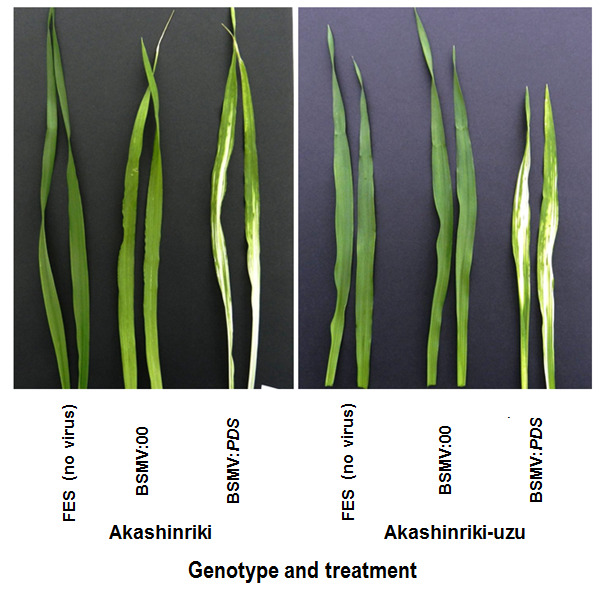


**Figure S3** Effect of virus-induced gene silencing (VIGS) of phytoene desature (PDS) on the phenotype of leaaves of barley cultivar Akashinriki and its uzu derivative. Codes: FES (no virus), just FES buffer used for viral application, BSMV:00, empty BSMV vector (negative control), BSMV:PDS, BSMV containing a construct targeting PDS for silencing. Silencing of PDS led to bleaching of barley leaves.
