# Supplementary material for: Plant disease resistance is augmented in uzu barley lines modified in the brassinosteroid receptor BRI1
Source: BMC Plant Biol. 2014 Aug 20;14:227. doi: 10.1186/s12870-014-0227-1 (PMC4158134; doi:10.1186/s12870-014-0227-1)
Supplement: Additional file 4: Table S3. — Primers used for real time quantitative RT-PCR analysis. Table S4. Primers used for VIGS. [file 12870_2014_227_MOESM4_ESM.doc]

**SUPPLEMENTARY TABLES**

**Plant disease resistance is augmented in uzu barley lines modified in the brassinosteroid receptor BRI1**

Shahin S. Ali^1,4©†^, Lokanadha R. Gunupuru^1†^, G.B. Sunil Kumar^1^, Mojibur Khan^1±^, Steve Scofield^2^, Paul Nicholson^3^ and Fiona M. Doohan^1©^

^1^Molecular Plant-Microbe Interactions Laboratory, School of Biology and Environmental Science, University College Dublin, Dublin 4, Ireland.

^2^USDA-ARS, Crop Production and Pest Control Research Unit and Purdue University, Department of Agronomy, 915 West Street, West Lafayette, IN 47907-2054, USA

^3^Dept. of Crop Genetics, John Innes Centre, Norwich Research Park, Norwich NR4 7UH, UK.

^4^SPCL, USDA/ARS Beltsville Agricultural Research Center, MD, USA.

±Present address: Institute of Advanced Study in Science and Technology, Guwahati -35, India.

^†^Equal contributors

©Corresponding author Email: shahinsharif.ali@gmail.com, fiona.doohan@ucd.ie

Fax: 0035317161102

Phone: 0035317162248

**Table S3** Primers used for quantitative RT-PCR analysis

| **Affymetrix**  **Probe ID.*^a^*** | **Probe annotation** | **Forward primer (5΄- 3΄)** | **Reverse primer (5΄- 3΄)** |
| --- | --- | --- | --- |
| Contig2170 | Pathogen-induced protein WIR1A | TGAGATCAAAGCGTCAGTCG | GCATCAAGGCCATTTAATCC |
| Contig2210 | Pathogenesis-related protein 1 | GACTACGGCTCCAACACCTG | TGACGTGCATCACGGTTAGT |
| Contig14570 | CER1 protein | CTATGCGATGACTCCTGCAA | CACTGTGTCTCCGCACTCAT |
| Contig8067 | Cellulose synthase-like protein | ACTATCGCGTTGCTCAACCT | CTGCCTTTGTCCTTCCTCAG |
| Contig3198 | Glycine-rich cell wall structural protein | GGCAGGAAGTGGAAGTGAAG | GGCACCACAACAGAGAAGTG |
| Contig7854 | BZR1 homolog in barley | AGGACGAGGAGCTGATGCTA | GCCGAACCCTCTCTAGCTCT |
| Contig5258 | XET homolog | GTCAGGAAGGAGCACACCAT | AAGCACGCATGAGCATACAG |
| Contig3160 | PHYB activation tagged suppressor 1 protein, BAS1 | TTTCTCATGGGGCAGTGAAT | GAATGGAAGTTACACCCTTGC |
| AB109215.1  Contig7854 | HvBri1  Brassinazole-resistant 2,BES1 | CAACGATGCTCAAGGTGATG  AGCCCTACCTCCTCTTCATTCC | CCGGTGGTCATCTTCCTAATCATGGTAGGATGGGAGTTGCA |
| Contig127_s | α-tubulin | GCATGGAGGAGGGAGAGTTC | CCAGGAGGCAGGCTCTAGTAC |
| Primers were design using the Primer3 software (version 0.4.0; http://frodo.wi.mit.edu/primer3/). Primer specificity was confirmed by melting curve analyses of real-time RT-PCR products (81 cycles of 55°C for 30s).  *^a^* Affymetrix Probes ID used in the microarray analysis were obtained from PLEX data base (http://www.plexdb.org) and the sequence was used for design of primers. | | | |

**Table S4** Primers used for VIGS

| **VIGS primer name** | **Forward primer (5΄- 3΄)** | **Reverse primer (5΄- 3΄)** |
| --- | --- | --- |
| HvBRI1:A | CGATTAATTAAGCGGAGGCAGAAGAATGA | CGACCCGGGGTCACCCTGGCCACTCAC |
| HvBRI1:B | CGATTAATTAAGTGAGTGGCCAGGGTGAC | CGACCCGGGTGGATGATGTGCGGAATG |
| HvBRi1:RT | CAACGATGCTCAAGGTGATG | CCGGTGGTCATCTTCCTAAT |

Primers were design using the Primer3 software (version 0.4.0; http://frodo.wi.mit.edu/primer3/).
